# Radiographic changes in midfoot alignment after hallux valgus correction with and without concomitant second tarsometatarsal arthrodesis: a comparative study

**DOI:** 10.1007/s00402-026-06471-5

**Published:** 2026-08-17

**Authors:** Jahyung Kim, Min Gyu Kyung, Yaelynn An, Nah Yon Kim, Kyoung Min Lee, Dong Yeon Lee

**Affiliations:** 1 Department of Orthopedic Surgery, Seoul National University Hospital, Seoul, Korea, Republic of; 2 Department of Orthopedic Surgery, Seoul National University College of Medicine, Seoul, Korea, Republic of; 3 Department of Orthopedic Surgery, Kyung Hee University Hospital at Gangdong, Seoul, Korea, Republic of; 4 Department of Orthopedic Surgery, Kyung Hee University College of Medicine, Seoul, Korea, Republic of; 5 Department of Orthopedic Surgery, Seoul National University Bundang Hospital, Seongnam-si, Korea, Republic of

**Keywords:** Hallux valgus, Medial column alignment, Second tarsometatarsal joint arthrodesis, Medial longitudinal arch, Transverse arch

## Abstract

**Introduction:**

Second tarsometatarsal (TMT) joint osteoarthritis occurs in a subset of patients with hallux valgus (HV). While second TMT arthrodesis is performed to address symptomatic arthritis, its association with midfoot alignment changes during HV correction remains unclear. This study aimed to compare changes in radiographic arch parameters between patients undergoing HV correction with and without concomitant second TMT arthrodesis.

**Materials and methods:**

This retrospective comparative study included 73 feet with HV and medial column malalignment (lateral Meary’s angle > 10°). The TMT group (*n* = 37) underwent HV correction combined with second TMT arthrodesis for symptomatic osteoarthritis, while the control group (*n* = 36) underwent isolated HV correction. These patients were matched to achieve comparable baseline characteristics. Radiographic parameters of the transverse arch (M2–M5 angle, C1–M2 distance) and medial column alignment (lateral Meary’s angle) were evaluated preoperatively and at a minimum 1-year follow-up. Functional outcomes were assessed as a secondary measure.

**Results:**

The TMT group demonstrated greater changes in transverse arch parameters compared with the control group. The M2–M5 angle decreased by 2.1° (95% CI, 1.4° to 2.7°) in the TMT group, whereas no significant change was observed in controls (−0.2°; 95% CI, −0.7° to 0.4°). The C1–M2 distance changed by 0.7 mm (95% CI, 0.5 to 0.9) in the TMT group and −0.2 mm (95% CI, − 0.3 to −0.1) in controls. Medial column alignment also changed to a greater extent in the TMT group, with a reduction in lateral Meary’s angle of 6.4° (95% CI, 5.0° to 7.9°), compared with 0.7° (95% CI, −0.7° to 2.1°) in controls, demonstrating a significant between-group difference. Both groups showed improvement in functional outcomes, with largely comparable postoperative scores.

**Conclusion:**

Second TMT arthrodesis performed during HV correction was associated with greater changes in transverse and medial arch parameters compared with HV correction alone. Functional outcomes were generally comparable between groups, although the clinical significance of the radiographic differences remains uncertain.

**Level of evidence:**

Level III, Therapeutic Study.

## Introduction

The first tarsometatarsal (TMT) joint is known to be a keystone for medial column stability, with isolated fusion reported to restore the medial longitudinal arch alignment [1, 2]. However, in patients with hallux valgus (HV), degenerative changes often extend beyond the first ray. End-stage osteoarthritis of the second TMT joint occurs in approximately 10% of HV cases and typically requires arthrodesis when symptomatic [3, 4]. Conservative management, including footwear modification and orthotic intervention, is commonly attempted prior to surgical correction in patients with HV, with the aim of redistributing plantar load and supporting the medial column [5]. Although second TMT arthrodesis is primarily performed to address localized pain from arthritis, its association with midfoot alignment changes in patients undergoing HV correction remains incompletely understood.

Schmidt et al. recently demonstrated on weight-bearing CT that transverse arch collapse is most pronounced between the medial cuneiform and second metatarsal base, designating this region as a biomechanical “hinge” linking the transverse and medial arches [6]. This observation suggests that the medial cuneiform-second metatarsal complex may contribute to overall midfoot structural configuration. Accordingly, stabilization of the second TMT joint during HV correction may be associated with changes in transverse or medial longitudinal arch parameters, which have not been clearly established. In addition, because HV correction itself can influence foot alignment, the independent contribution of second TMT arthrodesis remains difficult to determine [7].

The purpose of this study was to compare changes in radiographic arch parameters between patients undergoing HV correction with concomitant second tarsometatarsal (TMT) arthrodesis and those undergoing HV correction alone in the setting of medial column malalignment. Functional outcomes were evaluated as a secondary measure. We aimed to determine whether the addition of second TMT arthrodesis is associated with greater changes in transverse and medial arch alignment. We hypothesized that patients undergoing second TMT arthrodesis demonstrate greater radiographic changes in arch parameters compared with those treated with isolated HV correction.

## Methods

### Patients

This retrospective comparative study was performed between January 2015 and December 2023 at an urban tertiary-care referral center. Patients who underwent surgical correction for HV deformity at the institution by one senior surgeon were reviewed. From this cohort, HV patients demonstrating radiographic evidence of medial column malalignment were identified, defined as a lateral Meary’s angle (LMA) > 10° [8, 9]. Patients were excluded if they were < 18 years old, had less than one year of follow-up after the surgery, history of prior foot surgery (including hallux valgus revision), rheumatoid arthritis, hallux rigidus, or neuromuscular disease. We then identified cases that showed end-stage osteoarthritis of the second tarsometatarsal joint on pre-operative radiographs and severe, localized pain over the joint. These patients underwent concomitant second TMT joint arthrodesis based on clinical indication. The indication for second TMT arthrodesis was based on symptomatic osteoarthritis, which may reflect a different disease state compared with patients undergoing isolated HV correction. Those with radiographic non-union of the TMT joint fusion at the final review were excluded. As a result, 37 patients were included in the analysis and considered the TMT group.

**Fig. 1:**
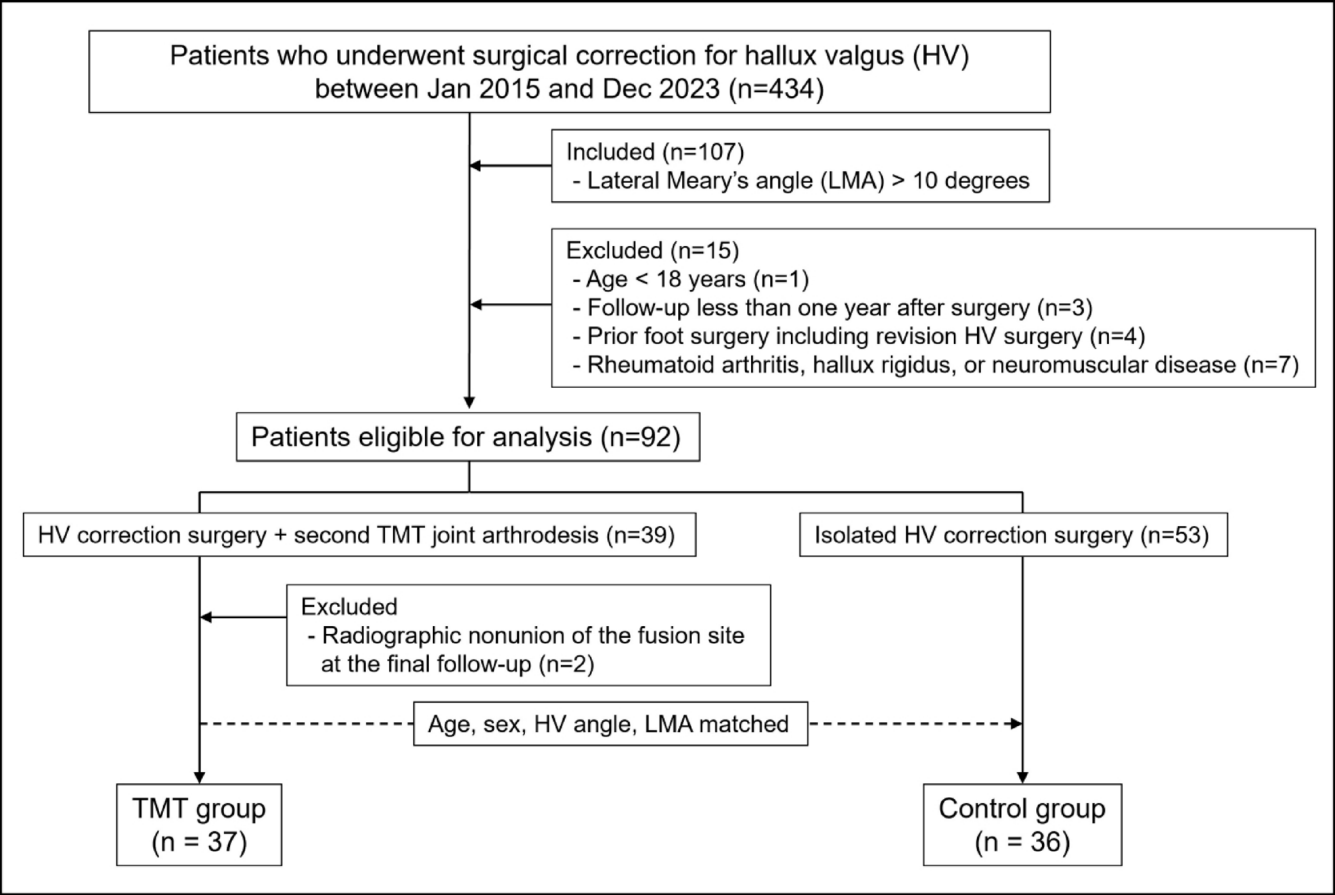
Flow diagram illustrating the patient selection process *HV* hallux valgus, *TMT* tarsometatarsal, *LMA* lateral Meary’s angle

For the control group, patients who underwent isolated HV correction in the presence of medial column malalignment, but without clinical indication for second TMT osteoarthritis, were identified. From the eligible control cohort, patients were selected to achieve group-level comparability with the TMT group in terms of age, sex, preoperative HVA, and LMA. A priori power analysis using G*Power (v3.1.9.7, two-tailed t-test, α = 0.05, effect size d = 0.5) determined that at least 27 patients per group were required to achieve 80% power [10]. After matching, 36 patients met the criteria and constituted the control group (Fig. 1).

### Operative procedures

All operations were performed by a single senior surgeon. HV correction employed a modified 90° distal chevron osteotomy with medial capsulotomy, Akin osteotomy, and first-web–space release. The distal chevron osteotomy was fixed with a single cortical screw, and the Akin osteotomy was fixed with a staple [11]. Second TMT fusion used a dorsal longitudinal approach, with meticulous joint preparation and fixation using a dorsal plate and screws. No adjunct procedures specifically intended to correct hindfoot deformity or global flatfoot alignment, including calcaneal osteotomy or subtalar fusion, were performed in either group. Post-operative care was identical, with suture/K-wire removal at 2 weeks, protective brace until 4 weeks, and full weight-bearing in regular footwear by 8 weeks.

### Radiographic measurements

**Fig. 2:**
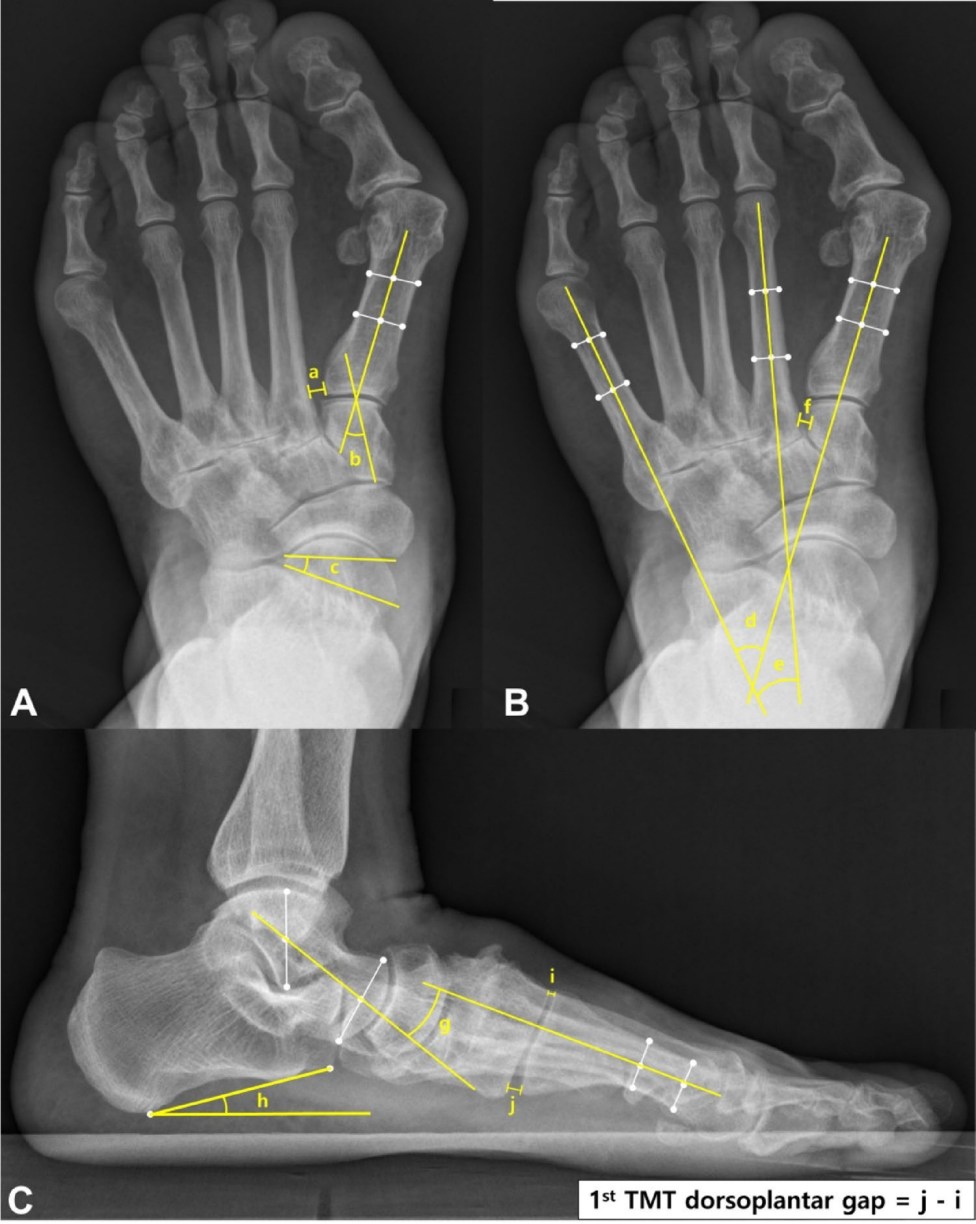
Radiographic measurements on weight-bearing foot radiograph A. First-second metatarsal distance (**a**), first metatarsal–medial cuneiform angle (**b**), talonavicular coverage angle (**c**) B. First-fifth intermetatarsal angle (**d**), second-fifth intermetatarsal angle (**e**), medial cuneiform to second metatarsal base distance (**f**) C. Lateral Meary’s angle (**g**), calcaneal pitch angle (**h**), 1 st TMT dorsoplantar gap (**j–i**). (*TMT* tarsometatarsal)

Weight-bearing anteroposterior and lateral radiographs obtained pre-operatively and at final follow-up were reviewed in PACS software (INFINITT, Seoul) by two fellowship-trained orthopedic surgeons (with 9 and 10 years of experience), with any discrepancies resolved by consensus. Hallux valgus correction was quantified with the HVA and the IMA [12]. Medial column alignment was characterized using established sagittal and midfoot parameters, including the first–second metatarsal distance, the talonavicular coverage angle, the LMA, and the calcaneal-pitch angle [8, 9, 13]. The degree of transverse arch collapse was assessed with three complementary indices. First, the first to fifth intermetatarsal angle (M1-M5 angle) was measured as the angle between the longitudinal axes of the first and fifth metatarsal shafts, with a larger angle signifying forefoot widening and flattening of the transverse arch [14]. Second, the second to fifth intermetatarsal angle (M2-M5 angle), which is formed by the second and fifth metatarsal shafts, was obtained. Because it excludes the first ray, it is less affected by hallux valgus varus drift and is considered a purer surrogate for transverse arch integrity [15]. Finally, the medial cuneiform to second metatarsal base distance (C1-M2 distance), defined as the shortest linear distance between the most medial cortex of the medial cuneiform and that of the second metatarsal base, was recorded; larger values reflect greater collapse of the apex of the transverse arch at the second TMT joint to medial cuneiform complex [6, 16]. First-ray instability under load was evaluated with the first metatarsal–medial cuneiform (M1–C1) angle and the first-TMT joint dorsoplantar gap (difference between the dorsal and plantar TMT joint spaces) (Fig. 2) [17]. All measurements were performed using standardized anatomical landmarks based on previously validated methods.

### Functional outcomes

Patient-reported outcome measures (PROMs) were assessed preoperatively and at final follow-up as a secondary measure to evaluate overall clinical improvement, using the Foot and Ankle Outcome Score (FAOS), American Orthoapedic Foot and Ankle Society (AOFAS) forefoot score, and visual-analog scale (VAS) for pain (0–10). [18, 19] FAOS was divided into five categories, including pain, symptoms, activities of daily living, sport and recreation, and quality of life (QOL) subdomains. Minimal clinically important difference (MCID) thresholds were defined based on validated studies: FAOS Pain 9.5, ADL 11.7, Sports/Recreation 8.7, and QOL 5.0 points [20]; FAOS-Symptoms 5.0 points [21]; AOFAS 7.9 points [22]; and VAS pain 2.0 points [23]. These MCIDs were used to help interpret the clinical significance of observed changes.

### Statistical analysis

Inter-observer reliability for radiographic measurements was assessed with a two-way random effects intraclass correlation coefficient (ICC, absolute agreement, single measures). Using Bonnett’s approximation, 15 paired observations were sufficient to achieve an ICC target of 0.90 with a 95% confidence-interval (CI) width of 0.20 [24].

Normality was checked with the Shapiro-Wilk test. Within-group pre-/post changes were analyzed with paired t-tests; between-group differences were compared with independent t-tests. Results are expressed as mean ± SD. The primary analysis focused on comparing changes in radiographic parameters between groups. Functional outcomes were analyzed descriptively and compared between groups as a second measure. All analyses were performed using R version 3.6.3 (R Foundation for Statistical Computing, Vienna, Austria), and a P value < 0.05 was considered statistically significant.

## Results

### Demographics

Baseline demographic characteristics were comparable between the two groups, with no differences in mean age (69.8 ± 5.6 vs. 68.7 ± 5.7 years, *P* > 0.15), BMI (25.7 ± 3.7 kg/m² vs. 24.6 ± 2.7 kg/m², *P* > 0.15), and mean follow-up duration (38.7 ± 27.5 months vs. 42.9 ± 29.2 months, *P* = 0.531). In addition, baseline radiographic parameters representing the HV deformity and medial column alignment demonstrated similar severity between groups, indicating comparable baseline deformity characteristics between the TMT and control groups (Table 1).

**Table 1:** Patient characteristics

	TMT group	Control group	*P*-value
	(*N* = 37)	(*N* = 36)	
Age (yrs)	69.8 ± 5.6	68.7 ± 5.7	0.389
BMI (kg/m^2^)	25.7 ± 3.7	24.6 ± 2.7	0.176
Sex (Female/Male)	36/1	33/3	0.588
Side (Right/Left)	13/24	18/18	0.295
Preoperative radiographic parameters			
Hallux valgus angle (°)	40.0 ± 8.5	39.0 ± 5.8	0.570
Intermetatarsal angle (°)	16.0 ± 2.8	15.4 ± 1.7	0.262
Sesamoid position			0.513
Normal	0 (0%)	0 (0%)	
Mild	1 (2.7%)	1 (2.78%)	
Moderate	5 (13.51%)	2 (5.56%)	
Severe	31 (83.78%)	33 (91.67%)	
Lateral Meary’s angle (°)	16.1 ± 5.6	16.4 ± 6.2	0.795
Follow-up duration (months)	38.7 ± 27.5	42.9 ± 29.2	0.531

*BMI* body mass index, *TMT* tarsometatarsal

### Changes in radiographic parameters

All radiographic parameters demonstrated excellent interobserver reliability (ICCs 0.91–0.99 preoperatively, 0.95–0.99 postoperatively) (Table 2).

**Table 2:** Interobserver reliability of radiographic measurements

	Preoperative			Postoperative		
	ICC	95% CI	*P*-value	ICC	95% CI	*P*-value
1 st −2nd metatarsal distance	0.934	0.896–0.958	< 0.001	0.947	0.917–0.967	< 0.001
Talonavicular coverage angle	0.924	0.881–0.952	< 0.001	0.979	0.966–0.987	< 0.001
Lateral Meary’s angle	0.993	0.986–0.996	< 0.001	0.978	0.959–0.988	< 0.001
Calcaneal pitch angle	0.987	0.975–0.993	< 0.001	0.990	0.980–0.994	< 0.001
M1-C1 angle (°)	0.965	0.944–0.978	< 0.001	0.965	0.944–0.978	< 0.001
1 st TMT joint dorsoplantar gap	0.991	0.984–0.996	< 0.001	0.980	0.961–0.990	< 0.001
M1-M5 angle (°)	0.949	0.919–0.968	< 0.001	0.948	0.908–0.970	< 0.001
M2-M5 angle (°)	0.938	0.903–0.961	< 0.001	0.962	0.941–0.976	< 0.001
C1-M2 distance (mm)	0.913	0.859–0.946	< 0.001	0.946	0.916–0.966	< 0.001

*ICC* intraclass correlation coefficient, *CI* confidence interval, *M1-M5 angle* first-fifth intermetatarsal angle, *M2-M5 angle* second-fifth intermetatarsal angle, *C1-M2 distance* medial cuneiform to second metatarsal base distance, *M1-C1 angle* first metatarsal-medial cuneiform angle, *TMT* tarsometatarsal

Baseline measures of medial longitudinal arch alignment and transverse arch width were comparable between groups (all *P* > 0.20). However, first-ray instability indices were greater in the TMT group: M1-C1 angle 25.1°±6.1 versus 21.1°±5.1 (*P* = 0.005) and first TMT dorsoplantar gap 2.1 ± 0.7 mm versus 1.3 ± 0.7 mm (*P* = 0.004), indicating a more unstable first ray despite similar HVA/IMA values. This difference suggests that the two groups may represent distinct clinical populations rather than comparable cohorts differentiated only by the addition of a procedure. Both groups achieved satisfactory HV corrections with no between-group differences in HVA or IMA correction (both *P* > 0.5) (Table 3).

**Table 3:** Changes in radiographic parameters and patient reported outcome measures (PROMs)

	TMT group				Control group						
	Preop	Postop	Mean diff	*P* ^1^	Preop	Postop	Mean Diff	*P* ^2^	*P* ^3^	*P* ^4^	*P* ^5^
Radiographic parameters											
Hallux valgus angle	40.0 ± 8.5	13.7 ± 6.7	26.3 (23.5, 29.1)	< 0.001	39.0 ± 5.8	13.9 ± 6.2	25.1 (22.7, 27.5)	< 0.001	0.57	0.887	0.518
Intermetatarsal angle	16.0 ± 2.8	6.5 ± 2.0	9.5 (8.6, 10.5)	< 0.001	15.4 ± 1.7	5.6 ± 2.3	9.8 (8.9, 10.7)	< 0.001	0.262	0.073	0.644
1st-2nd metatarsal distance	4.5 ± 1.2	3.3 ± 0.9	1.2 (0.8, 1.5)	< 0.001	4.0 ± 1.0	4.1 ± 1.1	0.0 (−0.4, 0.3)	0.844	0.162	0.003	< 0.001
Talonavicular coverage angle	26.1 ± 6.4	17.9 ± 6.3	8.2 (6.5, 9.9)	< 0.001	24.6 ± 7.1	23.8 ± 6.9	0.8 (−0.8, 2.4)	0.311	0.005	0.226	0.009
Lateral Meary’s angle	16.1 ± 5.6	9.6 ± 5.2	6.4 (5.0, 7.9)	< 0.001	16.4 ± 6.2	15.7 ± 5.9	0.7 (−0.7, 2.1)	0.316	0.899	< 0.001	< 0.001
Calcaneal pitch angle	16.2 ± 3.4	16.5 ± 4.7	−0.3 (−1.6, 1.1)	0.683	16.6 ± 2.8	16.6 ± 3.9	0.0 (−0.8, 0.8)	0.934	0.221	0.958	0.343
M1-C1 angle	25.1 ± 6.1	22.0 ± 4.8	3.1 (2.0, 4.2)	< 0.001	21.1 ± 5.1	20.6 ± 5.0	0.6 (−0.4, 1.5)	0.215	0.005	0.226	0.009
1 st TMT joint dorsoplantar gap	2.1 ± 0.7	1.1 ± 0.5	1.0 (0.8, 1.3)	< 0.001	1.3 ± 0.7	1.4 ± 0.8	−0.1 (−0.3, 0.1)	0.252	< 0.001	0.06	< 0.001
M1-M5 angle	35.3 ± 5.0	33.2 ± 5.1	2.1 (1.1, 3.2)	< 0.001	34.4 ± 4.1	35.0 ± 4.0	−0.5 (−1.2, 0.2)	0.135	0.414	0.1	< 0.001
M2-M5 angle	19.3 ± 4.2	17.2 ± 3.7	2.1 (1.4, 2.7)	< 0.001	19.5 ± 3.6	19.6 ± 4.2	−0.2 (−0.7, 0.4)	0.617	0.793	0.009	< 0.001
C1-M2 distance	4.6 ± 0.9	3.9 ± 1.0	0.7 (0.5, 0.9)	< 0.001	4.4 ± 0.8	4.6 ± 0.8	−0.2 (−0.3, −0.1)	0.005	0.375	< 0.001	< 0.001
PROMs											
FAOS Symptoms	73.4 ± 17.6	94.9 ± 7.9	−21.5 (−28.1, −14.9)	< 0.001	78.0 ± 17.1	91.5 ± 9.9	−13.5 (−19.9, −7.1)	< 0.001	0.243	0.113	0.082
FAOS Pain	59.4 ± 18.0	90.6 ± 11.2	−31.2 (−38.2, −24.2)	< 0.001	69.8 ± 20.7	92.3 ± 11.2	−22.2 (−30.2, −14.9)	< 0.001	0.016	0.504	0.097
FAOS ADL	72.7 ± 18.2	92.2 ± 8.8	−19.5 (−25.6, −13.3)	< 0.001	79.6 ± 15.9	92.8 ± 10.3	−13.3 (−19.8, −6.7)	< 0.001	0.108	0.765	0.164
FAOS Sports/Recreation	46.5 ± 26.0	61.5 ± 31.7	−15.0 (−28.6, −1.5)	0.031	57.1 ± 27.0	76.1 ± 25.2	−19.0 (−31.3, −6.8)	0.003	0.098	0.061	0.658
FAOS QOL	39.8 ± 22.8	69.2 ± 25.5	−29.4 (−40.3, −18.6)	< 0.001	39.4 ± 16.5	82.8 ± 22.0	−43.4 (−51.2, −35.7)	< 0.001	0.807	0.017	0.037
AOFAS	68.0 ± 18.1	85.2 ± 11.8	−17.2 (−24.9, −9.5)	< 0.001	69.9 ± 15.0	87.1 ± 11.3	−17.2 (−22.9, −11.5)	< 0.001	0.615	0.491	0.834
VAS	5.0 ± 2.1	1.7 ± 1.6	3.3 (2.5, 4.1)	< 0.001	3.6 ± 2.1	0.8 ± 1.3	2.7 (1.8, 3.6)	< 0.001	0.003	0.018	0.41

*P*^1^ : paired t-test between preoperative and postoperative variables in TMT group

*P*^2^ : paired t-test between preoperative and postoperative variables in Control group

*P*^3^ : independent t-test between TMT and control groups in preoperative variables

*P*^4^ : independent t-test between TMT and control groups in postoperative variables

*P*^5^ : independent t-test between TMT and control groups in pre- and postoperative differences

*TMT* tarsometatarsal, *M1-M5 angle* first-fifth intermetatarsal angle, *M2-M5 angle* second-fifth intermetatarsal angle, *C1-M2 distance* medial cuneiform to second metatarsal base distance, *M1-C1 angle* first metatarsal-medial cuneiform angle, *PROMs* patient-reported outcome measures, *FAOS* Foot and Ankle Outcome Score, *ADL* activities of daily living, *QOL* quality of life, *AOFAS* American Orthopaedic Foot and Ankle Society, *VAS* visual-analog scale

The TMT group demonstrated greater changes in medial column alignment parameters compared with the control group. First-second metatarsal distance decreased by 1.2 mm (95% CI, 0.8 to 1.5; *P* < 0.001) in the TMT group, whereas no significant change was observed in controls (0.0 mm; 95% CI, −0.4 to 0.3; *P* = 0.844), with a significant between-group difference in change. Talonavicular coverage angle decreased by 8.2° (95% CI, 6.5° to 9.9°; *P* < 0.001) in the TMT group but not in controls (0.8°; 95% CI, −0.8° to 2.4°; *P* = 0.311), and the between-group difference was significant. LMA decreased by 6.4° (95% CI, 5.0° to 7.9°; *P* < 0.001) in the TMT group, whereas minimal change was observed in controls (0.7°; 95% CI, −0.7° to 2.1°; *P* = 0.316), with a significant between-group difference in change. The TMT group also showed greater improvement in first-ray instability parameters, with M1-C1 angle decreasing by 3.1° (95% CI, 2.0° to 4.2°; *P* < 0.001) versus 0.6° (95% CI, − 0.4° to 1.5°; *P* = 0.215) in controls (*P* = 0.009), and first TMT dorsoplantar gap decreasing by 1.0 mm (95% CI, 0.8 to 1.3; *P* < 0.001) versus −0.1 mm (95% CI, − 0.3 to 0.1; *P* = 0.252) in controls.

All transverse arch parameters improved more significantly in the TMT group. M1-M5 angle decreased by 2.1° (95% CI, 1.1° to 3.2°; *P* < 0.001) in the TMT group, whereas it changed by −0.5° (95% CI, − 1.2° to 0.2°; *P* = 0.135) in controls. M2-M5 angle decreased by 2.1° (95% CI, 1.4° to 2.7°; *P* < 0.001) in the TMT group, whereas it changed by −0.2° (95% CI, −0.7° to 0.4°; *P* = 0.617) in controls. C1-M2 distance changed by 0.7 mm (95% CI, 0.5 to 0.9; *P* < 0.001) in the TMT group and by −0.2 mm (95% CI, − 0.3 to −0.1; *P* = 0.005).

### Functional outcomes

Preoperatively, the TMT group reported greater pain than the control group, with VAS 5.0 ± 2.1 versus 3.6 ± 2.1 (*P* = 0.003) and FAOS-Pain 59.4 ± 18.0 versus 69.8 ± 20.7 (*P* = 0.016). Both groups demonstrated improvement across all PROMs following surgery. Between-group comparisons showed no differences in the magnitude of change for any PROM except FAOS-QOL. FAOS-QOL improved by 29.4 ± 22.1 points in the TMT group versus 43.4 ± 18.7 points in controls (*P* = 0.037). All other sub-scales (FAOS-Pain, Symptoms, ADL, Sports/Recreation), AOFAS scores, and VAS pain showed similar changes between groups (all *P* > 0.10). At final follow-up, PROMs were largely comparable between groups, with two exceptions. FAOS-QOL remained lower in the TMT group (69.2 ± 25.5 vs. 82.8 ± 22.0; *P* = 0.017), and VAS pain was higher (1.7 ± 1.6 vs. 0.8 ± 1.3; *P* = 0.018). No significant differences were observed in other PROMs (all *P* > 0.10).

### Illustrative case

**Fig. 3:**
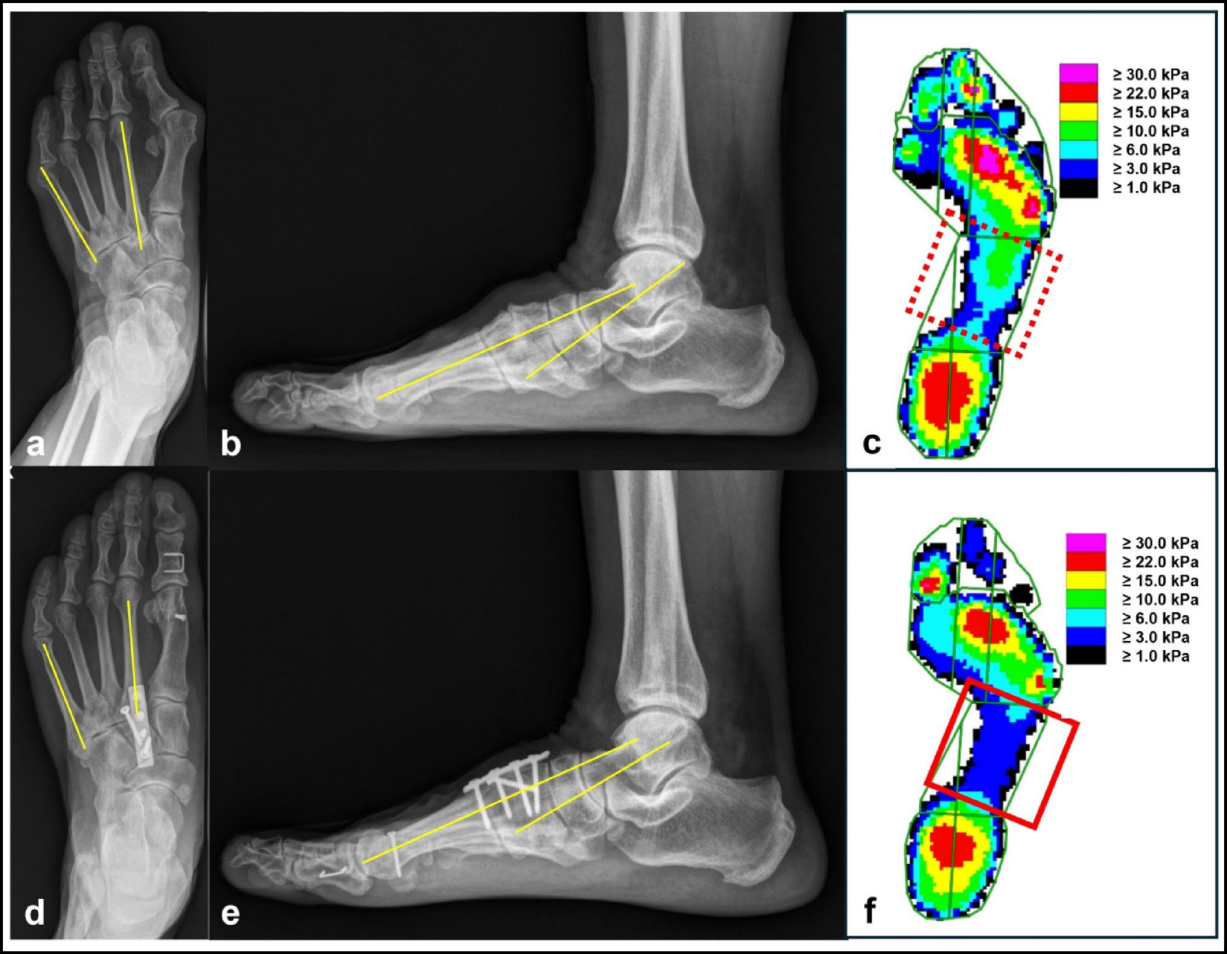
An illustrative case of a 63-year-old woman with hallux valgus and symptomatic second TMT osteoarthritis treated with combined hallux valgus correction and second TMT arthrodesis. **a**–**b** Preoperative radiographs demonstrate increased transverse arch width, with an M2–M5 angle of 17.6°, and sagittal-plane medial column malalignment (lateral Meary’s angle 17.7°). **c** Pedobarographic analysis demonstrates increased midfoot plantar pressure. **d**–**e** Postoperative radiographs demonstrate reduced transverse arch width (M2–M5 angle 14.0°) and improved medial column alignment (lateral Meary’s angle 2.4°). **f** Postoperative plantar pressure mapping demonstrates reduced midfoot loading, corresponding with changes in radiographic alignment. Yellow lines represent M2-M5 angle (**a** and **d**) and lateral Meary’s angle (**b** and **e**); red boxes and dotted lines indicate regions of midfoot pressure change (**c** and **f**)

A representative case is presented in Fig. 3 to illustrate the radiographic and plantar pressure changes following combined HV correction and second TMT joint arthrodesis. Pedobarographic analysis demonstrated a reduction in midfoot plantar pressure, which corresponded with changes in radiographic alignment.

## Discussion

This study addresses a clinical scenario encountered in patients undergoing HV correction with concomitant second TMT osteoarthritis and medial column malalignment. In this retrospective analysis of 73 patients, we observed that patients undergoing second TMT arthrodesis demonstrated greater changes in both transverse and medial longitudinal arch parameters compared with those undergoing HV correction alone. These findings should be interpreted as describing differences in radiographic changes between groups. Both groups demonstrated improvement in patient-reported outcomes following surgery.

Patients undergoing second TMT arthrodesis showed greater changes in transverse arch parameters, including reductions in the M2-M5 angle and C1-M2 distance. These findings indicate that transverse arch parameters changed to a greater extent in patients undergoing second TMT arthrodesis compared with those undergoing HV correction alone. In this context, Schmidt et al. described medial cuneiform-second metatarsal region as a structural “hinge” within the transverse arch based on weight-bearing CT analysis [6]. Our findings are consistent with this concept, in that radiographic changes were observed at this region following surgery. However, because both HV correction and second TMT arthrodesis were performed, the extent to which these changes are attributable to second TMT arthrodesis alone cannot be determined. Therefore, these results should be interpreted as describing radiographic changes observed in this cohort, rather than indicating a specific biomechanical mechanism.

The TMT group demonstrated a greater reduction in LMA compared with minimal change in the control group. This finding indicates that medial column alignment parameters changed to a greater extent in patients undergoing second TMT arthrodesis. Shima et al. reported favorable clinical and radiographic outcomes after combined HV correction and lesser tarsometatarsal arthrodesis in a small case series, but longitudinal arch alignment, including the LMA, remained largely unchanged [4]. In contrast, the present comparative cohort demonstrated a measurable change in LMA relative to the control group. Because HV correction itself can influence midfoot alignment, these findings should be interpreted with caution when considering the role of second TMT arthrodesis.

Although the two groups were matched for HV deformity and baseline medial column alignment, the TMT group demonstrated significantly greater first-ray instability preoperatively than the control group. This difference indicates that the TMT group likely represents a distinct clinical population with more advanced medial column pathology, rather than a directly comparable cohort distinguished only by the addition of a procedure. This may have influenced both surgical decision-making and radiographic outcomes. First-ray instability has been associated with altered load distribution across the forefoot, including increased loading of the second ray [25]. In this context, the presence of second TMT osteoarthritis in the TMT group may reflect a different stage or pattern of disease rather than an isolated local pathology [26]. Therefore, the observed radiographic changes in this study should be interpreted with consideration of these baseline differences. Further prospective studies are needed to better define the relationship between first-ray instability, second TMT joint degeneration, and surgical outcomes.

Both groups demonstrated improvement in PROMs following surgery, despite the TMT group presenting with worse baseline pain (VAS 5.0 vs. 3.6, *P* = 0.003). The largely comparable improvements in most functional outcomes between groups suggest that the addition of second TMT arthrodesis did not adversely affect overall clinical recovery. However, given that the TMT group had symptomatic osteoarthritis, improvements in PROMs are likely influenced by pain relief from the affected joint, and thus may not be fully attributable to changes in radiographic alignment. Nevertheless, some differences in patient-reported outcomes persisted at final follow-up. Specifically, the TMT group demonstrated lower FAOS-QOL scores and higher VAS pain compared with the control group. This discrepancy likely reflects differences in baseline disease characteristics rather than the effect of the surgical procedure itself. Patients in the TMT group had symptomatic second TMT osteoarthritis requiring arthrodesis, suggesting a more advanced or complex disease state, which may contribute to persistent symptoms or perceived limitations despite radiographic improvement. In addition, patient-reported quality of life is influenced by multiple factors beyond structural alignment and may not directly correlate with radiographic changes. Although statistically significant radiographic differences were observed, these did not consistently translate into clinically meaningful differences in patient-reported outcomes based on established MCID thresholds.

This study has several limitations. First, the retrospective design limits causal inference. In addition, the inclusion of patients with symptomatic second TMT osteoarthritis introduces confounding by indication. Because the surgical indication for second TMT osteoarthritis is inherently linked to disease severity, residual confounding cannot be fully eliminated, thereby limiting causal interpretation of the findings. Second, baseline differences in first-ray instability and the fact that HV correction itself can influence midfoot alignment make it difficult to isolate the independent effect of second TMT arthrodesis. Third, the single-surgeon, single-center design may limit generalizability, although it ensured consistency in surgical technique and postoperative protocols. Specifically, all procedures were performed using a modified distal chevron technique. Surgical approaches that more directly address medial column instability, such as proximal opening wedge osteotomy or Lapidus arthrodesis, may interact differently with second TMT fusion, and these findings may not be directly applicable to other techniques. Fourth, medial column malalignment was defined using radiographic parameters without comprehensive assessment of hindfoot alignment or peritalar stability, and therefore these findings should be interpreted within this context. In addition, the LMA represents a continuous parameter without a universally accepted cutoff. Although a threshold of 10° was used in this study, higher thresholds have been described to reflect more advanced deformity rather than to define the presence of malalignment. Accordingly, this threshold should be interpreted as a practical definition to capture clinically relevant deformity. Finally, the minimum follow-up period of 1 year may be insufficient to fully evaluate the progression of medial column instability, the development of adjacent joint degeneration, and the long-term functional impact of the procedure.

## Conclusions

This study showed that patients undergoing second TMT arthrodesis during HV correction exhibited greater changes in transverse and medial arch parameters compared with those undergoing HV correction alone. These findings suggest that midfoot alignment may change to a greater extent when second TMT arthrodesis is performed in this clinical setting. Despite worse baseline pain in the TMT group, functional outcomes improved in both groups and were largely comparable at final follow-up. However, the clinical importance of these radiographic changes remains uncertain, as they did not consistently translate into improved patient-reported outcomes. Therefore, these findings should not be interpreted as supporting second TMT arthrodesis solely for alignment correction in the absence of symptomatic TMT pathology. Overall, our findings highlight the importance of evaluating second TMT pathology in patients with HV and medial column malalignment, and further studies are needed to clarify the relationship between midfoot alignment changes and clinical outcomes.

## Data Availability

No datasets were generated or analysed during the current study.
